# Prakruti genomics and prameha-proclivity: relevance to metabolic syndrome

**DOI:** 10.1186/1755-8166-7-S1-I3

**Published:** 2014-01-21

**Authors:** Ashok DB Vaidya

**Affiliations:** 1 ICMR-Advanced Centre of Reverse Pharmacology in Traditional Medicine, Kasturba Health Society, Mumbai, India

## 

Ayurveda, an art-science of a healthy way of life and a system of medicine, is being practiced in India for several millennia. Despite the remarkable description of diabetes (*Madhumeha*) and its precondition (*Prameha*), in Ayurveda, the current biomedical sciences stay generally uninformed of its rich concepts.

The proclivity factors for *Prameha* are: *Garbhakala Asatmyata* (intrauterine influence), *Shaithilya* (poor muscle and adipose firmness), *Meda asarata* (dysfunctional adipocytes), *Madhur Agraha* (sweet tooth), *Kapha chaya* (accumulation of sero-mucoids), *Kapha prakopa* (aggravation of biofilms), *Mansavahasrotadushti* (hepatomuscular infiltration), *Meda Vriddhi* (adiposity), *Avyayama* (distaste for exertion), *Divasvapna* (daytime sleep), *Medovahasrotadushti* (adipose tissue inflammation), *Kleda Dushti* (excess of extracellular fluid) etc. The relative significance of each of these factors will depend on the individual’s *Prakruti*. Genetic determinants of these factors have to be investigated.

The profiling of *Prakruti* in patients with diabetes has been studied by several groups. In a study from the Central Research Institute (Ayurveda), Jaipur, the frequency of *kapha/* with *vata/pitta* was much more (64 %) than pure *pitta/kapha*/with *vata* (36 %). The response to the treatment with *Nisha amalaki* was also better in patients of *kapha prakruti.* The other study from the Banaras Hindu University showed a strong correlation of blood glucose response to exercise with prakruti. In another study from BHU, an association was found between the type 2 diabetes mellitus and 5, 10-methylene tetrafolate reductase MTHFR C677T. The CT genotype is protective. The study did not show any association of genotypes with the prakruti. MTHFR C677T gene polymorphism, common in the Chinese population, presents a genetic risk factor for diabetic nephropathy in type 2 diabetic patients. So there is also a need to consider Prakruti genomics for assessing the risk, onset and severity of complications in diabetes. Reverse Pharmacolgy coupled with Ayurgenomics can be applied to detect and measure the variation in drug response correlates vis-à-vis Prakruti.

In the development of obesity, variants of more than three dozen genes are identified. For the type 2 diabetes, there are estimates of around hundred genes being involved; most of them are not identified at present. But it is being realized that it would be too simplistic to expect genomics to provide all the answers. Besides proteomics and metabolomics, we need a paradigm shift to consider the problem of proclivity to diabetes from the perspective of human biology at the bedside. Prakruti, Pathya and Ahar-Vihar of Ayurveda offer such bedside opportunities. These interactions, at present, are formidable challenges to the dominant reductionist paradigms in genomics and systems biology. However, personalized prevention and management of the metabolic syndrome can influence its resolution or progression to diabetes mellitus if one can identify the sets and subsets of *prakruti* with objective clusters of genomic, proteomic or metabolic markers; this would emerge by research at the interface of Ayurveda and Modern Biomedicine.

